# Artificial Intelligence for Automatic Monitoring of Respiratory Health Conditions in Smart Swine Farming

**DOI:** 10.3390/ani13111860

**Published:** 2023-06-02

**Authors:** Eddiemar B. Lagua, Hong-Seok Mun, Keiven Mark B. Ampode, Veasna Chem, Young-Hwa Kim, Chul-Ju Yang

**Affiliations:** 1 Animal Nutrition and Feed Science Laboratory, Department of Animal Science and Technology, Sunchon National University, Suncheon 57922, Republic of Korea; eddiemarlagua@gmail.com (E.B.L.); mhs88828@nate.com (H.-S.M.); keivenmarkampode@sksu.edu.ph (K.M.B.A.); veasna.chem09@gmail.com (V.C.); 2 Interdisciplinary Program in IT-Bio Convergence System (BK21 Plus), Sunchon National University, 255 Jungangno, Suncheon 57922, Republic of Korea; 3 Department of Multimedia Engineering, Sunchon National University, Suncheon 57922, Republic of Korea; 4 Department of Animal Science, College of Agriculture, Sultan Kudarat State University, Tacurong City 9800, Philippines; 5 Interdisciplinary Program in IT-Bio Convergence System (BK21 Plus), Chonnam National University, Gwangju 61186, Republic of Korea; yhkim8760@naver.com

**Keywords:** artificial intelligence, PRDC, respiratory disease, smart farming, swine

## Abstract

**Simple Summary:**

This paper provides a review of recent studies exploring the application of artificial intelligence (AI) in the early detection and monitoring of respiratory disease in swine, emphasizing the significance of early detection for preventing economic losses. The studies primarily focus on utilizing coughing sounds as a feature in disease recognition, comparing different AI models and methodologies. A commercially available AI system that integrates temperature and humidity sensors with audio technologies for respiratory health monitoring through cough-sound identification is also assessed. However, the limitations of the current technology are identified, highlighting the need for further advancements to develop smarter AI solutions for swine respiratory health monitoring.

**Abstract:**

Porcine respiratory disease complex is an economically important disease in the swine industry. Early detection of the disease is crucial for immediate response to the disease at the farm level to prevent and minimize the potential damage that it may cause. In this paper, recent studies on the application of artificial intelligence (AI) in the early detection and monitoring of respiratory disease in swine have been reviewed. Most of the studies used coughing sounds as a feature of respiratory disease. The performance of different models and the methodologies used for cough recognition using AI were reviewed and compared. An AI technology available in the market was also reviewed. The device uses audio technology that can monitor and evaluate the herd’s respiratory health status through cough-sound recognition and quantification. The device also has temperature and humidity sensors to monitor environmental conditions. It has an alarm system based on variations in coughing patterns and abrupt temperature changes. However, some limitations of the existing technology were identified. Substantial effort must be exerted to surmount the limitations to have a smarter AI technology for monitoring respiratory health status in swine.

## 1. Introduction

Porcine respiratory disease complex (PRDC) is one of the most economically important diseases in the swine industry globally [1]. This disease causes economic losses to the industry through increased mortality, increased production costs due to increased medication and measures to control the disease, increased condemnation in the abattoir, and reductions in growth performance [2]. PRDC is induced by a combination of environmental conditions such as temperature, dust, ammonia, and carbon dioxide and pathogens including porcine reproductive and respiratory syndrome virus (PRRS), porcine circovirus (PCV2) type 2, *Actinobacillus pleuropneumonia* (APP), *Mycoplasma hyopneumoniae* (MHP), *Mycoplasma hyorhinis* (MHR), *Pasteurella multocida* (PM), *Haemophilus parasuis* (HPS), etc. [3].

Early detection of the disease is crucial for the early deployment of control measures to prevent further economic losses [4]. In the conventional method, the disease diagnosis is conducted through clinical examination and necropsy of dead or euthanized animals and sample collection during farm visits. However, this method is stressful for the animals and expensive, and the result is not real-time which delays the appropriate intervention. Artificial intelligence (AI) has been used in smart livestock farming for many applications including animal welfare and health detection and monitoring [5,6,7]. AI can detect subtle changes in animals, making it more effective in the early detection of disease compared to the conventional method and without time limitations [8,9].

There are several review articles that have been published regarding the application of artificial intelligence in smart agriculture or in smart livestock farming. However, to the authors’ knowledge, there is no study that specifically discusses the application of AI intelligence in respiratory disease detection and monitoring in swine. Therefore, this study was conducted. As the first topic of this paper, the authors discuss the importance of respiratory disease in the swine industry. Secondly, the authors review studies that were published between 2012 and 2022 about respiratory disease detection in swine using AI. In this section, the performance of different AI models and methodologies used is reviewed and compared. Thirdly, the AI technology available in the market is reviewed. Lastly, the authors discuss the limitations of the existing AI technology and synthesize recommendations for the future development of the technology.

## 2. Importance of PRDC in the Swine Industry

Global meat production in 2020 reached up to 337 million tons, and pork represented 33% of the total meat production, which makes it the second-largest source of meat among livestock species after poultry meat [10]. According to Komarek et al. [11], the global demand for livestock-derived foods is projected to increase by 38% from 2020 to 2050, driven by population growth and rising incomes in developing countries. It is a challenge for the swine industry to meet the demand for good quality and cheap pork in a situation where global warming is worsening and the prevalence of infectious diseases is increasing.

PRDC is caused by complex factors, including either or a combination of viral (including swine influenza, PRRS, and PCV2) and/or bacterial (including APP, MHP, MHR, PM, and HPS) factors, and is influenced by environmental conditions such as temperature, dust, ammonia, and carbon dioxide [3]. Viral pathogens are the primary causative agent of PRDC that can induce severe lesions and suppress the immune system and attract secondary infection and coinfection with bacteria [12]. The prevalence of PRRS and PCV2 in top pork-producing countries was investigated. In China, the prevalence of PRRS and PCV2 from 2017 to 2021 was 52.04% and 29.75%, respectively [13]; high prevalence was recorded in 56 commercial farms in South Korea with 85.70% and 51.80%, respectively [14]; and the prevalence of PRRS in 3 regions of Ontario, Canada, ranged from 17.0% to 48% [15]. In an investigation conducted in Brazil, only 0.11% (15/12,841) of serum samples from commercial pigs, imported boars, and wild and feral pigs were positive for PRRS. The prevalence of such PRDC-associated pathogens varies from country to country. However, this statistic highlights the importance of surveillance programs to prevent entry and control the spread of the disease.

PRDC is a major concern for the swine industry, resulting in significant economic losses due to decreased pig productivity, increased mortality, increased condemnation at slaughter, and increased medication costs resulting in high production costs [1]. A financial analysis by Calderón Díaz et al. [2] on Irish farrow-to-finish farms showed the negative economic impact of PRDC. The annual net profit was reduced by 14.6%, 12.8%, and 41.0% on farms infected with PRRS, swine influenza, and MHP, respectively. The treatment and control of PRDC are challenging and expensive, making prevention crucial for the swine industry. To minimize the impact of PRDC, swine producers implement strict biosecurity measures to prevent the entry of disease from other farms or within the farm, vaccination, quarantine and acclimatization, proper hygiene practices, and early disease detection [1,16,17]. Effective PRDC control and management are important for the sustainability and profitability of the swine industry.

## 3. Artificial Intelligence for Respiratory Disease Detection

Fever, inappetence, lethargy, coughing, sneezing, and dyspnea are common clinical signs of respiratory disease in pigs [18,19]. However, coughing is the most evident symptom and is used for the assessment of the respiratory health condition of the herd [20]. In developing AI for monitoring respiratory health status in pigs, coughing sounds are mostly used as a feature. Eye temperature, ear base temperature, respiration rate, and heart rate can also be used as features using computer vision [1,21]. However, the application of cameras for monitoring respiratory health status is still in the experimental stage. In this paper, the authors mainly reviewed studies that used audio technologies for respiratory disease detection.

### 3.1. Development of AI Technology for Cough Recognition

Pig cough recognition studies using machine learning and deep learning involve four steps: data acquisition, preprocessing, feature extraction, and classification. Other studies involved the fusion step for fusing two or more features after feature extraction [22,23,24,25]. As shown in Table 1, the performance of the AI models for cough recognition in terms of accuracy, recall, precision, and F1 score could reach more than 97%. This suggests that AI technology can be used for the automatic monitoring of respiratory health status with high reliability. Nevertheless, differences in performance were observed, and there is more opportunity for improvement. The AI model’s performance regarding cough recognition is affected by the methodologies used, particularly in the sound data preprocessing, feature extraction, and classification steps [22,25,26,27]. These are discussed in the next subsections. 

Types of data acquisition tools (sound sensors) can affect sound quality because of noise and eventually degrade the performance of the model in the recognition of sounds [28]. Therefore, the specification of the sound sensor or microphone must be considered in developing an AI device for cough recognition. The devices used in the studies for sound data collection were unidirectional cardioid microphone [22,23,24,29], omnidirectional electret microphone [27,30], M260C microphone [25], digital camcorder [26], CCTV camera with sound sensors [31,32], recording pen [33], and sound sensors fixed in ear tags [34,35,36,37]. Monitoring the respiratory health of an individual animal is feasible using ear tags with sensors. However, it is not cost effective compared to group monitoring. In addition, battery life and proneness to damage are a great concern [36].

As shown in Table 2, most of the studies that have been conducted have focused on the improvement of the performance of the AI model in cough recognition through the application and development of different architectures in the feature extraction and classification steps. Other studies have proposed the use of body-conducted sound for the early detection of respiratory disease [34,35,37]. Wang et al. [27] investigated the association between animal sounds and house air quality. Coughing sounds vary according to the type of infection. AI can distinguish the differences and detect specific infections, for example, MHP, PRRS, and postweaning multisystemic wasting syndrome (PMWS) in pigs [31,38]. Though most of the studies used cough as a feature to detect respiratory disease, sneezing can also be used in the early detection of influenza infection [30].

Wu et al. [39] proposed using vision and audio in respiratory disease detection using a CCTV camera with sound sensors. Eye temperature, ear base temperature, respiration rate, and heart rate can be used as features in respiratory disease detection using an infrared camera and RGB camera [1,21]. The respiration rate and the heart rate can also be measured using a piezoelectric sensor fixed in the ear tag [34,35,37]. Each sensor has some limitations regarding its application in disease detection. The incorporation of two or more sensors is necessary to compensate for the limitations of the individual. Therefore, the ideal AI technology in disease detection is a hybrid.

**Table 1:** Performance of different AI models in cough recognition.

Extraction Technique	Classification Technique	Sound Dataset (N)	Accuracy	Recall	Precision	F1	Literature
Acoustic (*RMS, ZCR, MFCC, Centroid, Flatness, Bandwidth, Rolloff, & Chroma*) & visual (*LBP & HOG*)	SVM	3157	96.45	97.33	96.83	97.08	Ji et al. [22]
MFCC + ΔMFCC	Improved SE-DenseNet-121	1445	93.80	98.60	97.00	97.80	Song et al. [25]
Acoustic (*RMS, ZCR, MFCC, Centroid, Flatness, Bandwidth, Rolloff, Contrast & Flux*) & deep feature	SVM	2546	97.35	96.51	98.41	97.46	Shen et al. [24]
STFT	Fine-tuned AlexNet	4480	95.40	96.80	95.50	96.20	Yin et al. [29]
MFCC–CNN	SVM	4551	96.68	97.72	96.81	97.26	Shen et al. [23]
	Softmax		95.82	95.51	97.33	96.41	
PMFCC	SVM	200	95.00				Wang et al. [27]
DNS	CNN					96.57	Choi et al. [26]
	SVM					95.15	
	KNN					93.74	
	C4.5					85.10	
MFCC	SVDD/SRC		94.00	92.00	90.80		Chung et al. [31]
Average			95.56	96.35	96.10	95.28	

RMS: root-mean-square energy; ZCR: zero-crossing rate; MFCC: mel-frequency cepstral coefficient; ΔMFCC: first-order difference of MFCC; PMFCC: principal MFCC; LBP: local binary pattern; HOG: histogram of oriented gradients; STFT: short-time Fourier transform; DNS: dominant neighborhood structure; CNN: convolutional neural network; SVM: support vector machine; KNN: k-nearest neighbors; SVDD: support vector data description; SRC: sparse representation classifier.

### 3.2. Feature Extraction and Fusion Techniques

Datasets contain a large amount of irrelevant information. In the feature extraction step, variables that significantly represent the cough-sound signal are selected and combined into a multidimensional feature vector. This step is the most crucial step for sound recognition [40,41]. In speech and sound recognition, the mel-frequency cepstral coefficient (MFCC) is the most popular feature used. Accordingly, the MFCC maps the linear spectrum of the sound signal onto the nonlinear mel spectrum and analyzes the spectrum according to human hearing [25].

In field conditions, background noises (non-cough sounds) are abundant and inevitable and can degrade the quality of cough sounds and affect model recognition performance. The method to improve model performance is to preprocess the sound data to enhance the quality of the cough sounds using background noise filtering [24,25,27,33,38] and by training the model with background noise data [42]. Feature extraction techniques can also solve this problem. One of the reasons for the popularity of the MFCC is its anti-noise capability [25,27,31]. However, this statement contradicts the statement of Ji et al. [22] based on the result of Zhao et al. [43]. Furthermore, the method proposed by Choi et al. [26] using the dominant neighborhood structure (DNS) algorithm as an extraction method has shown superior performance in a noisy environment. It is also much better than the MFCC feature. Furthermore, Wang et al. [27] obtained an average recognition rate of 95% after reducing the dimensionality of the MFCC using principal component analysis (PCA), which is better than Chung et al.’s [31] result using conventional MFCC features.

Much effort has been made in research to improve the precision of pig cough recognition. Most of the studies focused on feature extraction techniques. To overcome the limitation of the individual feature, fusion of two or more features to create a new feature has proven to improve cough-sound recognition. Shen et al. [23] fused the MFCC and convolutional neural network (CNN) features to obtain MFCC–CNN, which has shown significantly better performance compared to using MFCC features. Similarly, performance was also improved when fusing all acoustic features (root-mean-square energy or RMS, zero-crossing rate or ZCR, MFCC, spectral centroid, spectral flatness, spectral bandwidth, spectral roll-off, and contrast and flux) and deep features [24]. Furthermore, the fusion of acoustic features and visual features (local binary pattern or LBP and histogram of gradient or HOG) also has superior performance compared to individual features [22]. In the study of Song et al. [25], the combination of MFCC and ΔMFCC has a better performance compared to the MFCC alone. The ΔMFCC is the first-order difference of the MFCC, which is the relationship between two adjacent frames of pig sound signals.

### 3.3. Classification Techniques

Classifier architectures are classified into traditional machine learning classifiers and deep neural networks (DNN) [44]. A CNN is a popular deep neural network architecture that is inspired by neurons in the human brain. It has been widely applied in different fields like computer vision, face recognition, and sound and speech recognition [45,46,47]. In human health, it can be used in cough recognition [48,49] and in the detection of COVID-19 infection through cough and breathing sounds [50]. Choi et al. [26] compared CNN with traditional machine learning classifiers like support vector machine (SVM), k-nearest neighbors (KNN), and C4.5 in pig sound recognition under various noise conditions. CNN outperformed the other classifier architectures. Unlike the traditional classifiers, CNN can further extract deeper abstract features for classification and can learn in time and frequency simultaneously [51].

Nowadays, there are several CNN architectures that have been presented that differ in strengths and performance [52]. Modification of the architectures has been studied to improve classification performance. To improve the cough recognition performance of a deep learning algorithm, Song et al. [25] came up with an improved DenseNet architecture which they called SE-DenseNet-121. The SE-DenseNet-21 has an improved dense block module and is embedded with a Squeeze-and-Excitation Network (SENet) attention module. The purpose of the SENet is to optimize the extraction of relevant features and ignore irrelevant features. To improve the performance of conventional AlexNet, Yin et al. [29] combined it with softmax architecture and found that it has better performance in cough-sound classification compared to the probabilistic neural network (PNN). The softmax classifier uses logistic regression statistics in classification [53]. It has been widely used in the field of computer vision, especially in deep learning [54]. Similarly, TransformerCNN (a fusion of CNN and an encoder part of Transformer) has better performance in classifying different pig sounds compared to other classifier architectures [55].

**Table 2:** Summary of studies that involve smart technologies in respiratory disease detection.

Technique	Sensors	Number of Pigs Used (Head)	Objective	Findings	Literature
Vision	TIR camera (FLIR AX8) & RGB video camera (Raspberry Pi Camera Module V2.1)	76 (9-week-old)	Utilizing computer-based methods, thermal infrared and conventional images are employed to gauge alterations in the temperature of pigs (through eye- and ear-based measurements) as well as their heart and respiration rates.	✔ Infected pigs showed higher temperature and heart rate than healthy pigs. ✔ Respiration rate showed less difference between infected and healthy pigs. ✔ The use of computer vision techniques can furnish significant and actionable information regarding physiological alterations that may signify initial indications of respiratory infection in pigs.	Joquera-Chavez et al. [1]
	TIR camera (FLIR Duo^®^ Pro R)	46 (9-week-old)	To assess the effectiveness of utilizing computer-based methods with RGB and thermal infrared imagery in measuring the heart rate and respiration rate of pigs and to explore the possibility of utilizing remote assessments of eye temperature, heart rate, and respiration rate as a means of identifying early indications of respiratory diseases in growing pigs that are group-housed and free-moving within a commercial piggery.	✔ The remotely obtained heart rate and respiration rate were compared with the measures obtained with standard methods, showing positive correlations (r = 0.61–0.66; *p* < 0.05). ✔ Clear variations in remotely obtained eye temperature and heart rate were observed between healthy and sick pigs two days prior to the appearance of clinical indications.	Joquera-Chavez et al. [21]
Audio	Piezoelectric sensor & MEMS microphone	4 (5-week-old)	To propose a wireless system to record body-conducted sounds of pigs individually and a method of analysis for the early detection of respiratory diseases in infected pigs.	✔ Zero-crossing & MFCC values of body-conducted sound were significant before and after PRRS inoculation. ✔ Heart rate and respiratory rate can be measured indirectly through body-conducted sound which can be used for disease detection.	Narusawa et al. [35]
	Piezoelectric sensor & MEMS microphone	4 (5-week-old)	To develop a system for early detection of respiratory diseases in pigs utilizing body-conducted sound.	✔ Zero-crossing & MFCC values of body-conducted sound were significant before and after PRRS inoculation. ✔ Respiratory and heart sounds can be effectively extracted by recording BCS from the tip of ear.	Cheng et al. [34]
	Piezoelectric sensor & MEMS microphone	4 (5-week-old)	To develop a system for early detection of respiratory diseases in pigs utilizing body-conducted sound.	✔ Zero-crossing & MFCC values of body-conducted sound were significant before and after PRRS inoculation. ✔ Heart rate and respiratory rate can be measured indirectly through body-conducted sound which can be used for disease detection.	Tsuchiya et al. [37]
	Directional cardioid microphone	NI (commercial farm)	To improve the recognition accuracy of pig coughs using a new fusion feature (MFCC–CNN).	✔ Classifiers that utilized the MFCC–CNN feature demonstrated considerably superior performance in comparison to those that employed the MFCC feature. ✔ Fusing 55 and 45 adjacent frames resulted in the best performance for the softmax and SVM classifiers, respectively.	Shen et al. [23]
	Digital camcorder & CCTV with an audio sensor	36 (25–35 kg)	To propose an efficient data-mining solution for the detection and recognition of pig wasting diseases using sound data in audio surveillance systems.	✔ A combination of MFCC and SVDD can automatically detect pig wasting diseases using cough sounds at an accuracy level of 94%. ✔ The SRC classified pig wasting diseases into PMWS, PRRS virus, and MHP with an average accuracy of 91.0%.	Chung et al. [31]
	Omnidirectional electret microphone	280 (8.5-week-old; 25 kg)	To identify the relationship between animal sounds and air quality of animals’ living environment.	✔ Cough sounds from weaners differ significantly under different air quality conditions. ✔ The developed model had recognition accuracy of 95%.	Wang et al. [27]
	Recording pen (Mrobo M66)	10 (age & weight NI)	To propose DNN–HMM model to construct an acoustic model for continuous pig cough-sound recognition.	✔ The DNN–HMM-based acoustic model for continuous pig cough-sound recognition was stable and reliable.	Zhao et al. [33]
	Sound sensor (MAX9814) in ear tag	2 (18 kg)	To propose a remote monitoring tool for the objective measurement of some behavioral indicators that may help in assessing health and welfare status—namely, posture, gait, vocalization, and external temperature.	✔ Sensors can be successfully incorporated in ear tag. ✔ No coughing recorded during the trial.	Pandey et al. [36]
	Audio sensor	36 (25–35 kg)	To propose an economical and lightweight sound-based pig anomaly detection system that can be applicable even in small-scale farms.	✔ The results of the abnormality identification experiment demonstrated an F1 score of 0.947. ✔ The execution time of the abnormality identification algorithm on the TX-2 board was 0.253 s, which was 0.220 s faster than the basic MnasNet model.	Hong et al. [38]
	Omnidirectional electret microphones (Panasonic, WM-61A)	16 (age & weight NI)	To examine the correlations between the frequency of sneezing and various strains of influenza virus in domestic pigs.	✔ The classification performance of the automatic sneeze detector was observed to be close to 100%. ✔ The infection of certain swine influenza virus strains can be detected a few days after the infection by analyzing the frequency of induced sneeze.	Mito et al. [30]
	Microphone (LIQI LM320E, Cardioid electret microphone)	128 (17-week-old; 60 kg)	To propose a feature fusion method by combining acoustic and deep features from audio segments.	✔ The proposed acoustic and deep feature fusion achieved 97.35% accuracy for pig cough recognition. ✔ CQT is more suitable for sound recognition in a pig housing environment than traditional linear STFT.	Shen et al. [24]
	M260C Microphone Array with six SPA1687LR5H-1microphone components	6 (age & weight NI)	To develop SE-DenseNet-121 model to recognize pig cough sounds.	✔ The rate of recognition accuracy, recall, precision, and F1 score of the SE-DenseNet-121 recognition model for pig cough sounds were 93.8%, 98.6%, 97%, and 97.8%, respectively.	Song et al. [25]
	Microphone (LIQILM320E, Cardioid electret microphone)	128 (17-week-old; 60 kg)	To propose a novel feature fusion method that fuses acoustic and visual features to achieve an enhanced pig cough recognition rate.	✔ The fused acoustic features (Acoustic) combined with LBP and HOG (A-LH) achieved 96.45% pig cough accuracy.	Ji et al. [22]
	Digital camcorder (JVC GR-DVL520A)	36 (25–30 kg)	To propose a noise-robust system for the classification of sound data.	✔ The proposed method can be used to classify sound events in a cost-efficient manner while maintaining high levels of accuracy even in the presence of environmental noise.	Choi et al. [26]
	Microphone (LIQI LM 320ECardioid electret microphone)	NI (commercial farm)	To provide a highly accurate pig cough recognition method for the respiratory disease alarm system using fine-tuned AlexNet model and spectrogram feature.	✔ The proposed algorithm significantly outperforms the other algorithms—cough and overall recognition accuracies reach 96.8% and 95.4%, respectively, with 96.2% F1 score achieved.	Yin et al. [29]
Vision & audio	CCTV with an audio sensor	NI (commercial farm)	To propose a method to detect wasting disease automatically using both audio and video data.	✔ The Motion History Image-Based method can discriminate the shaking motion of coughing from other movement motions with careful motion pattern analysis.	Kim et al. [32]
Others	Recording pen (Lenovo B610)	NI (commercial farm)	To propose a pig sound classification method based on the dual role of signal spectrum and speech.	✔ An accuracy of 93.39% was achieved for the pig speech classification task.	Wu et al. [39]
	Recording pen (Lenovo B610)	NI (commercial farm)	To propose a sound classification model called TransformerCNN, which combines the advantages of CNN spatial feature representation and the Transformer sequence coding to form a powerful global feature perception and local feature extraction capability.	✔ The scores for domestic pig sound recognition accuracy, AUC, and recall were 96.05%, 98.37%, and 90.52%, respectively.	Liao et al. [55]

NI: not indicated; TIR: thermal infrared; RGB: red–green–blue; MEMS: micro-electromechanical systems; BCS: body-conducted sound; SRC: sparse representation classifier; MFCC: mel-frequency cepstral coefficient; CNN: convolutional neural network; SVDD: support vector data description; DNN–HMM: deep neural network–hidden Markov model; CQT: constant-Q transform; STFT: short-time Fourier transform; LBP: local binary pattern; HOG (A-LH): acoustic combined with LBP (local binary pattern) and HOG (histogram of oriented gradients); AUC: area under the curve; PMWS: postweaning multisystemic wasting syndrome; PRRS: porcine reproductive and respiratory syndrome; MHP: *Mycoplasma hyopneumoniae*.

SVM is the most popular traditional machine learning classifier used for sound applications [44], and it is the common classifier used in the reviewed studies for cough- and non-cough-sound classification. It is more robust compared to deep neural network classifiers for a few datasets available for training [24], for example, that in Wang et al. [27] with only 200 sound samples. The principle of SVM is to generate a hyperplane that separates the data into two classes [56]. The functionality of SVM is dependent on its kernel function, which should be for its application [57]. The commonly used kernel functions are linear, Gaussian radial basis function (RBF), and polynomial [58]. Wang et al. [27] used the RBF kernel in pig cough recognition because of its easy design, good generalization, and robust tolerance to noises. More than one kernel can be used in SVM. For example, Shen et al. [24] and Ji et al. [22] used RBF for the non-linear model and linear kernel model in cough recognition.

Other studies used KNN, C4.5, and random forest (RF) in cough recognition. In KNN, the trained data are grouped into subsets, and it classifies the new data on the basis of their values and assigns them to the nearest neighbor [59]. C4.5 is based on a decision-tree classification algorithm [60]. The RF is similar to C4.5. However, the RF generates multiple trees for decision-making [61]. Compared to SVM, these classifiers are inferior in performance in pig cough and non-cough-sound classification [22,23,26]. Shen et al. [23] compared SVM and the softmax in cough recognition. They found that SVM is superior to the softmax. However, this contradicts other studies, especially regarding computer vision [54,62].

Among the studies reviewed, no existing models reached more than 99% performance. That means that there is still room for improvement. The robustness of the model is still the priority concern. However, researchers should also prioritize the speed of recognition without compensating performance. 

### 3.4. Performance Evaluation Metrics

Many metrics are proposed for AI model algorithm performance evaluation. However, the most common metrics used for cough recognition in the reviewed studies are based on the confusion matrix. These are accuracy, precision, recall or sensitivity, specificity, and F1 score (Table 3). Accuracy is the proportion of correctly classified coughing-sound samples to the total number of the dataset. Accuracy alone is not a reliable parameter for evaluating the cough recognition model. It is sensitive to imbalanced datasets [63], which is not good if there are more non-cough-sound data than cough-sound data, or vice versa. A model can have high accuracy but a low level of recognition of cough sounds. 

Precision is the ratio of correctly classified cough-sound samples to the total samples classified as cough sounds. Recall is the ratio of correctly classified cough-sound samples to the total cough-sound dataset. Therefore, the higher the precision and recall, the better the model for recognizing cough-sound data. However, this does not mean that the model has a high recognition rate of non-cough sounds. An ideal AI technology should be able to distinguish cough sounds from non-cough sounds at a high rate. In this case, specificity is the matrix. Specificity is the ratio of correctly classified non-cough-sound samples to the total non-cough-sound dataset. Furthermore, high precision and recall do not coincide in a real scenario. The F1 score measures the balance of precision and recall, and, accordingly, it is a good candidate for a formal classifier quality evaluation metric [64]. In developing an AI model for cough recognition, it is important to consider all the metrics and not use them individually to be able to capture the strengths and weaknesses of the model.

## 4. Commercial Application

SoundTalks (SoundTalks NV, Leuven, Belgium) is a pioneering and popular AI product for automatic respiratory disease detection that is available on the market today. Additionally, new similar technologies developed in the United States by MASCO Technologies (City of Industry, CA, USA) and in China by iFLYTEK Co., Ltd. (Hefei, China) and Muyuan Foods Co., Ltd. (Nanyang, China). However, the authors have limited knowledge about these new technologies, and thus, the review focuses solely on SoundTalks. This review was conducted to understand the features and the limitations of the existing technology, providing a foundation for the future development of more precise smart technology. 

### 4.1. Features of SoundTalks

SoundTalks is an AI technology available on the market today that can automatically monitor the respiratory health status of herds (in the nursery to finishing stages) through cough-sound recognition and quantification. It has sensors for monitoring environmental conditions such as room temperature and relative humidity. It also has an alert system that signals the farmers about potential respiratory problems and abrupt temperature changes. 

**Figure 1:**
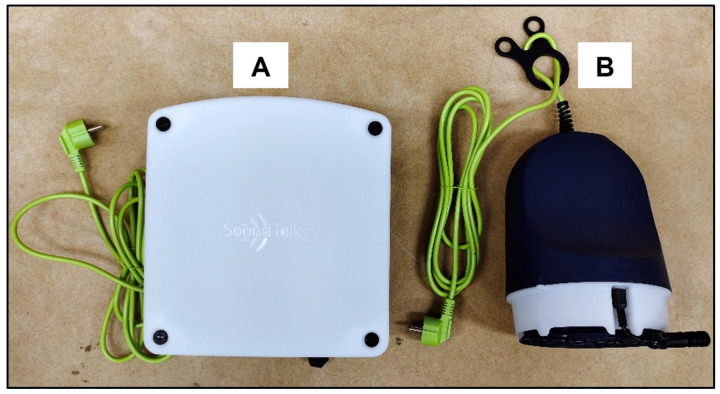
SoundTalks has two pieces of hardware: (**A**) the gateway and (**B**) the monitor.

This device has two main pieces of hardware: the monitor and the gateway (Figure 1). The sound sensor (microphone) and the environmental sensors are fixed to the monitor. The device also has LED lights for alert indication. It can gather sound data within a 10 m radius. All the data from the monitor are transferred to the gateway wirelessly. A plurality of monitors can be connected to the gateway not more than 30 m from the location of the gateway. The gateway is connected to a network via LAN. The function of the gateway is to connect and gather data from the monitor and send data to the AI cloud for data processing and analysis. Users can access their data online from every monitor through a PC or smartphone via a mobile app. A strong and undisrupted internet connection is essential for this technology to work optimally. 

The respiratory health status of the herd is presented as respiratory distress index (RDI). The RDI is the average number of coughs per head within 24 h [65]. Nowadays, the respiratory health status of the herd is presented as ReHS (respiratory health status), with values ranging from 0 to 100. An alert system is an important feature of health monitoring technology for notifying farmers of abnormalities within the farm. This device has a patented algorithm that uses the history of and variation in the respiratory health status of the herd for the calculation of the threshold as the basis of the alarm [65]. A green light represents a normal ReHS, which ranges from 60 to 100. Yellow and red alerts mean there is a potential and a high risk of a respiratory problem, respectively. These are equivalent to 40–59 and 0–39 ReHS, respectively. For thermal shock alerts, a yellow warning is issued when there is a ≥5 °C difference in temperature within a 6 h interval. Furthermore, a red alert is issued when there is a ≥8 °C difference. The alerts can be perceived through LED lights on the monitor and through their website.

### 4.2. Research Using SoundTalks

Several studies have been conducted using SoundTalks for automatic quantification of the respiratory health status of pigs because of its advantages and reliability. It has shown that the coughing frequencies recorded using manual and automatic quantifications of coughing have a similar trend [65,66]. However, manual quantification is time-consuming and not efficient in a field situation. Moreover, farmers are not present 24 h inside the house. Therefore, coughs are often not detected, particularly at night [67].

A study by Clavijo et al. [5] showed that this device can detect respiratory disease in MHP-infected pigs. The RDI value increased as more pigs tested positive for MHP. The first alert was observed 55 days post-infection (DPI), 27 days late from the first day of MHP detection in uninoculated pigs. Similarly, Poeta Silva et al. [66] found an increase in RDI value in MHP-infected pigs as the infection progressed. However, an alarm from the device was not observed during the study despite mortality being observed at 10 DPI, and the RDI value was lower compared to Clavijo et al. [5]. The population is the main factor in these differences because this device was designed to recognize cough sounds in large pig populations [65], suggesting that the alarm system in future AI technology should work in multiple populations and should be refined on the basis of the severity of infection for earlier detection.

Data generated from SoundTalks can be used to improve the management of the housing environment and respiratory disease in finishing pigs. Pessoa et al. [68] conducted a study that correlates coughing frequency and environmental conditions of healthy pigs. They found that coughing frequencies are affected by a high ammonia concentration and high ventilation rates. However, the coughing incidence and ammonia concentration during the study were low. Another study by Pessoa et al. [65] has found an association between lung lesions at slaughter and values of RDI toward the end of growing. Coughing episodes in the early stage of the growing period are not associated with the prevalence of lung lesions at slaughter. This could mean that intervention and treatment should be conducted once a coughing episode is observed in the late finishing stage to reduce condemnation at slaughter. 

**Figure 2:**
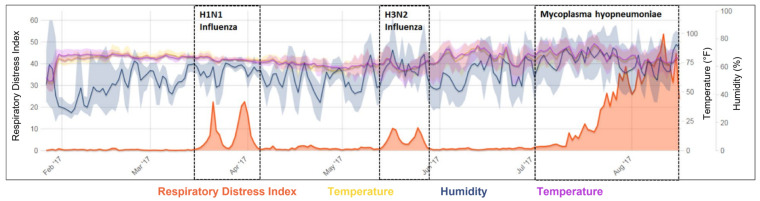
Chart showing Respiratory Disease Index (RDI), temperature, and humidity data from a wean-to-finish barn experiencing clinical episodes of influenza and *Mycoplasma* [69].

SoundTalks is not yet able to distinguish whether coughing is caused by pathogens or by environmental conditions. However, by thoroughly analyzing the patterns of RDI, PRDC-associated pathogens can potentially be distinguished. For example, as shown in Figure 2, the RDI patterns in influenza A virus-infected pigs had a distinct double-peaked shape, whereas the pattern associated with MHP showed a gradual increasing pattern [69]. Further study is recommended to compare the RDI/ReHS patterns of pigs infected with other PRDC-associated pathogens and environmental conditions. With this information, farmers will be given a tool for more timely, precise diagnosis and control of the disease. 

## 5. Existing Technology: Limitations and Opportunities for Improvement

In a decade, several studies have been conducted on AI’s application in livestock production. One application of AI is cough detection for monitoring the respiratory health status of swine. AI for cough detection has been implemented in commercial applications, and it has been found to have a great advantage in enhancing farming productivity and efficiency through improvements in decision-making for herd health management. However, the authors found some limitations of the existing technology that should be considered for the improvement and development of smarter technology.

### 5.1. Farm-Dependent Coughing Threshold

Early detection of a respiratory problem is crucial for controlling the disease to prevent further economic losses. Thus, an alarm system is a very important feature of technology for accurately and promptly alerting the user of an early respiratory problem. The existing technology has a patented algorithm that calculates the threshold as the basis of the alarm. This threshold is based on the history of and the variation in the respiratory health status of a specific room where a monitor is installed. This system has a possible drawback. For example, two farms with different coughing histories may have different thresholds, and once the farms are infected at the same rate, one room might not alarm (possibly the room with more coughing at the start). Thus, further research is recommended to generate a threshold value that is associated with the severity of infection. As stated, historical data were used for threshold calculation as the basis for the alarm. In the authors’ opinion, the algorithm was designed to detect acute respiratory disease. Is this algorithm able to detect chronic respiratory disease? This is something that needs to be explored. 

### 5.2. Population-Dependent Coughing Threshold

The existing technology is built to monitor the respiratory health status of a large population. Several studies found lower RDI values and no alert even in infected, small populations compared to large populations. The threshold for alarm should be associated with infection. Further research is recommended to generate a threshold value that is corrected by population.

### 5.3. Monitoring of Herd Respiratory Health Status

Studies using audio technology have shown great performance in detecting respiratory problems caused by pathogens or environmental conditions. However, the current technology can only evaluate the general health status of the herd in a specific room. Automatic recognition of individual pigs is essential for monitoring an individual’s health condition. Currently, the application and development of computer vision technology for individual pig recognition are still in the experimental stage, the cost of hardware being a notable limiting factor in its large-scale application. However, the integration of visual technology with audio technology can be a better technology for monitoring the health of the herd and the individual pig. In this system, the specific sick pig can be immediately recognized, and the health management will be more precise.

## 6. Conclusions

PRDC is a major concern for the swine industry that results in significant economic losses. Thus, early detection of and response to the disease at the farm level is essential for preventing and minimizing the potential damage that it may cause. Studies about the application of AI in respiratory health disease detection have shown its great advantage in swine farming. Respiratory diseases can be detected using AI for automatic cough-sound recognition and quantification with high sensitivity and precision. However, different AI models have demonstrated differences in performance. These differences might be due to methodologies and architectures used in data preprocessing, feature extraction, and classification. Additionally, the size and diversity of the dataset used for training are crucial for this part.

The AI technology available is commercially used audio technology that can monitor and evaluate the herd’s respiratory health status with temperature and humidity sensors to monitor environmental conditions. Moreover, some limitations of the existing technology were identified. The limitations are mainly regarding the sensitivity of the alarm system to coughing episode variations. The alarm system is farm-dependent and population-dependent. Lastly, it cannot detect a respiratory problem in an individual pig. Substantial effort must be exerted to surmount the limitations to have a smarter AI technology for monitoring respiratory health status in swine.

## Figures and Tables

**Table 3:** AI model performance metrics based on a confusion matrix.

Metrics	Definition	Formula
True Positive	The number of samples that are correctly classified as cough sounds	TP
True Negative	The number of samples that are correctly classified as non-cough sounds	TN
False Positive	The number of samples that are incorrectly classified as cough sounds	FP
False Negative	The number of samples that are incorrectly classified as non-cough sounds	FN
Accuracy	The ratio of correct predictions over the total number of dataset	$\frac{\mathrm{TP}+\mathrm{TN}}{\mathrm{TP}+\mathrm{TN}+\mathrm{FP}+\mathrm{FN}}$
Precision	The ratio of correctly classified cough-sound samples to the total samples classified as cough sounds	$\frac{\mathrm{TP}}{\mathrm{TP}+\mathrm{FP}}$
Recall	The ratio of correctly classified cough-sound samples to the total cough-sound dataset	$\frac{\mathrm{TP}}{\mathrm{TP}+\mathrm{FN}}$
Specificity	The ratio of correctly classified non-cough-sound samples to the total non-cough-sound dataset	$\frac{\mathrm{TN}}{\mathrm{FP}+\mathrm{TN}}$
F1 score	Combination of precision and recall	$2\times \frac{\mathrm{Precision}\;\times \;\mathrm{Recall}}{\mathrm{Precision}\;+\;\mathrm{Recall}}$

## Data Availability

Not applicable.
